# Enhancement of the Peroxidase-Like Activity of Iodine-Capped Gold Nanoparticles for the Colorimetric Detection of Biothiols

**DOI:** 10.3390/bios10090113

**Published:** 2020-09-01

**Authors:** Chia-Chen Chang, Tsz-Lian Hsu, Chie-Pein Chen, Chen-Yu Chen

**Affiliations:** 1 Department of Medical Biotechnology and Laboratory Science, Chang Gung University, Taoyuan City 333, Taiwan; 2 Department of Obstetrics and Gynecology, Mackay Memorial Hospital, Taipei City 104, Taiwan; elisaxha@gmail.com (T.-L.H.); cpchen@mmh.org.tw (C.-P.C.); 3 Department of Medical Research, MacKay Memorial Hospital, New Taipei City 251, Taiwan; 4 Department of Medicine, Mackay Medical College, New Taipei City 252, Taiwan

**Keywords:** colorimetric assay, catalytic gold nanoparticle, iodine, peroxidase-like activity

## Abstract

A colorimetric assay was developed for the detection of biothiols, based on the peroxidase-like activity of iodine-capped gold nanoparticles (AuNPs). These AuNPs show a synergetic effect in the form of peroxidase-mimicking activity at the interface of AuNPs, while free AuNPs and iodine alone have weak catalytic properties. Thus, iodine-capped AuNPs possess good intrinsic enzymatic activity and trigger the oxidation of 3,3’,5,5’-tetramethylbenzidine (TMB), leading to a change in color from colorless to yellow. When added to solution, biothiols, such as cysteine, strongly bind to the interface of AuNPs via gold-thiol bonds, inhibiting the catalytic activity of AuNPs, resulting in a decrease in oxidized TMB. Using this strategy, cysteine could be linearly determined, at a wide range of concentrations (0.5 to 20 μM), with a detection limit of 0.5 μM using UV-Vis spectroscopy. This method was applied for the detection of cysteine in diluted human urine.

## 1. Introduction

Enzymes are important and essential biomacromolecules in natural systems. They regulate various biochemical functions and active behavior with high selectivity and fast reaction rate. They also provide an efficient catalyst for use in research and industry applications without the need for harsh chemical environments, including in macromolecular syntheses, biofuel production, and biomolecular detection [1,2,3]. However, the practical use of natural enzymes is limited because of their low stability, short storage life, and high cost.

Since the fabrication of the first nanomaterial-based artificial enzyme mimicking peroxidase activity [4], the development of nanoenzymes has attracted considerable attention. Artificial nanoenzymes have several advantages over natural enzymes, including high stability against denaturing and simple storage conditions [5,6]. Accordingly, many nanomaterials, including noble metal-based nanostructures, transition-metal dichalcogenides, carbon-based nanostructures, and metal-organic frameworks have been designed to have intrinsic enzyme-like activities, such as peroxidase [7,8], catalase [9,10], oxidase [11,12], lactase [13], and ferroxidase [14,15]. In addition, some nanomaterials with multiple enzyme-like activities have been developed, such as porous Co_3_O_4_ nanoplates, which exert both peroxidase- and catalase-like activities [16]. Most reactions of nanozymes occur on the surface of nanomaterials. The modification of surface would thus change the catalytic activities. For example, the coating of DNA or other biomolecules have been found for inhibiting the activities of nanozymes [17,18]. However, in some cases, a modification would form a favorable environment to enhance or recover the activities of nanozymes [19,20,21]. For instance, phosphite was reported to recover the oxidase-like activity of nickel oxide nanoparticles [21].

Among nanomaterial-based colorimetric strategies, gold nanoparticles (AuNPs) are of great interest due to their unique physical and chemical properties [22]. AuNPs are known to be stabilized by electrostatic repulsion; aggregation-based AuNP colorimetric assays are widely used for the detection of protein [23,24,25], small molecule [26,27,28], and nucleic acid targets [29,30]. Moreover, such colorimetric assays are simply adaptable to a smartphone-based device without the use of costly instruments [31,32]. Recently, AuNPs were found to show intrinsic peroxidase-mimicking activity resulting from weakly bound reactive species on their surface, and have been used instead of peroxidases in enzyme immunoassays [33]. Taking into account the tunability, biocompatibility, and ease of synthesis of AuNPs, these nanoparticles are good candidates for designing nanosensors. Many researchers have focused on the regulation of the peroxidase-like activity of AuNPs for the detection of H_2_O_2_ and small molecules [34]. In addition, some functional molecules, such as heavy metal ions [35,36] and aptamers [37,38], have been implemented in the design of biosensors by tuning the catalytic capability of AuNPs. Nevertheless, the sensitivity of the AuNP-based colorimetric assay remains unsatisfactory due to the low catalytic activity of AuNPs.

**Scheme 1:**
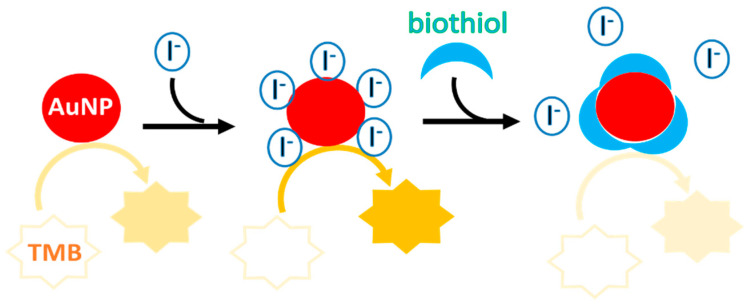
Schematic illustration of a colorimetric biosensing assay based on iodine-capped AuNPs as a peroxidase-like enzyme mimetic for the detection of biothiols.

To address the limitation imposed by this low catalytic activity, Huang et al. developed an assay for the colorimetric detection of mercury ions (Hg^2+^) after finding that Hg^2+^ could improve the catalytic activity of citrate-capped AuNPs [39,40]. Shah et al. found that adenosine triphosphate (ATP) markedly induced catalytic activity in AuNPs, but did not yet use ATP as a sensor for other molecules [41]. In a previous study, we found that iodide ions (I^−^) served as an etchant of two-dimensional gold nanotriangles for the sensing of antibiotics [42]. In the present study, I^−^ showed potential for use as an activity promoter to expand the peroxidase-like activity of AuNPs. We also found that the interfacial properties of AuNPs could be reversibly switched in response to biological stimuli. This behavior makes AuNPs an attractive candidate for establishing label-free colorimetric sensors. In this study, catalytic iodide-capped AuNPs were used as signal reporters to convert the binding reaction into a colorimetric response. The sensing principle is illustrated in Scheme 1. I^−^ ions are known to readily attach to the surface of gold through the chemisorption of iodide [43]. Thus, the catalytic ability of AuNPs was enhanced once I^−^ became attached to the surface of AuNPs via Au–I linkages. Conversely, upon the addition of biothiol molecules (cysteine as the model), I^−^ became displaced from the AuNP surfaces due to the Au–S bond being stronger than the Au–I linkage [44], resulting in the loss of the peroxidase-like activity of AuNPs. As the thiol group has a higher capability to gold, the approach developed in this work is, therefore, suitable for the identification of biothiol molecules.

## 2. Materials and Methods

### 2.1. Chemicals

The AuNP solution was supplied by BBI Solutions (Cardiff, UK). H_2_O_2_ (30%) was obtained from Fluka (AG, Buch, Switzerland). TMB stop solution was purchased from SeraCare (Milford, MA, USA). Sigma-Aldrich (St. Louis, MO, USA) supplied 3,3’,5,5’-tetramethylbenzidine (TMB), potassium iodide (KI), alanine (Ala), glutamine (Gln), glycine (Gly), proline (Pro), histidine (His), leucine (Leu), valine (Val), phenylalanine (Phe), tyrosine (Tyr), glutathione (GSH), homocysteine (Hcy), cystine (Cy), ascorbic acid (AA), and riboflavin (RF). All other chemical reagents were of analytical grade. Ultrapure water was utilized in this study.

### 2.2. Determination of Cysteine in an Aqueous Solution

Briefly, 10 μL of AuNP solution, 45 μL of ultrapure water, and 0.5 μL of I^−^ solution (4 mM) were mixed. After allowing them to react for 15 min, 40 μL of different concentrations of cysteine solution were added to the mixture. After 15 min, 2 μL of H_2_O_2_ (10 M) and 2.5 μL of TMB solution (6 mM) were added into the reaction solution. After 5 min, 5 μL of stop solution was added to the solution. The absorption spectra were measured at 450 nm after 2.5 min using a Sunrise ELISA plate reader (Tecan Austria GmbH, Salzburg, Austria).

To understand the catalytic state of AuNPs, the normalized absorbance was calculated as ΔA450/A450_0_ (ΔA450  = A450_0_ − A450_x_), where A450_0_ and A450_x_ were the absorbance intensities of the AuNP system before and after the addition of cysteine, respectively. The error bars were obtained by calculating the standard deviation of the normalized absorbance values and from three independent experiments in the same 96-well microplate.

### 2.3. Detection of Cysteine in Real Samples

Urine samples were collected from healthy volunteers. Prior to spiking, the addition of acetonitrile was used for protein precipitation [45,46]. Thus, 100 μL of urine subsamples were mixed with 300 μL of acetonitrile and vortexed for 1 min. The samples were then centrifuged at 12,000 rpm for 30 min at 4 °C to remove the protein matrix. Forty microliters of the resulting supernatants was added to 360 μL of cysteine standard solution. The absorbance was measured at 450 nm and used to determine the concentration of cysteine in urine.

## 3. Results and Discussion

### 3.1. Principle of the Colorimetric Cysteine Assay

**Figure 1:**
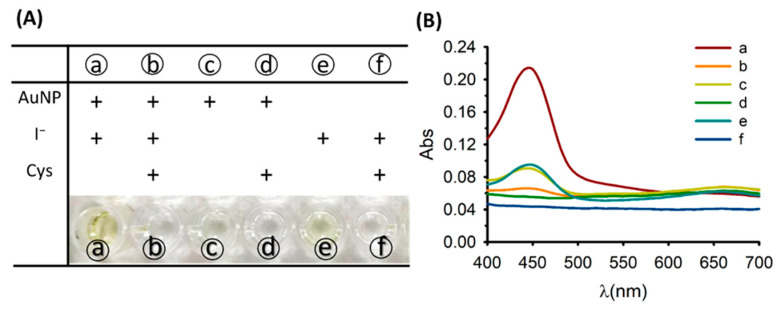
(**A**) Color changes of the solution under different conditions. The “+” indicates the presence of the corresponding substance. (**B**) Absorption spectra of the catalytic AuNP solution under different conditions. The final concentrations of I^−^ and cysteine were 5 μM and 50 μM, respectively.

The catalytic properties of AuNPs were investigated. The iodide ions were found to catalyze the oxidation of colorless TMB by H_2_O_2_, resulting in the generation of a yellow product that released colorimetric signals. In our study, a small reaction occurred in the presence of citrate-capped AuNPs (Figure 1Aⓒ) and iodide ions (Figure 1Aⓔ) alone, indicating their weak catalytic activity. In contrast, a mixture of AuNP and I^−^ was found to induce the oxidation of TMB by H_2_O_2_, resulting in a deep yellow color (Figure 1Aⓐ). Figure 1B compares the UV–vis spectra resulting from the addition of I^−^/AuNP, AuNP, and I^−^ under different conditions. No remarkable increase in absorption intensity at 450 nm was observed in the presence of AuNPs and I^−^ separately. However, when combined, a chromogenic reaction resulted in a marked increase in absorbance at 450 nm. In addition, the intensity of absorbance at 450 nm for I^−^/AuNP was higher than that for AuNP and I^−^ alone (Figure 1B), indicating the higher catalytic activity of I^−^/AuNP. The attachment of I^−^ onto the surface of AuNPs was confirmed using fluorescein isothiocyanate-capped AuNPs (FITC-AuNPs). FITC fluorescence was restored when I^−^ was added to the FITC-AuNP solution, indicating the replacement of FITC by I^−^ ions and the release of FITC from the gold surface (Figure S1 in Supplementary Materials). This is consistent with previous results that I^−^ is absorbed onto the AuNP surfaces [47,48,49]. As such, we ascribed the improvement in nano-enzymatic activity to the efficient synergetic catalytic effect at the interface of the AuNPs and the attached I^−^ ions. As shown in Figure 1, AuNPs alone or free I^−^ ions showed a lower catalytic activity toward TMB.

The interface of AuNPs could be reversibly changed from “active” to “inactive” by the addition of a biothiol species, such as cysteine, to the solution. Originally, I^−^ was adsorbed on the interface of the AuNP catalysts, thereby, enhancing their catalytic activity. However, upon the addition of cysteine, I^−^ responsively detached from the surface of AuNPs through thiol (cysteine) displacement. Consequently, the surface that was originally activated by I^−^ became passive, terminating the catalytic reaction of AuNPs, and thus generating a change in color from yellow to colorless (Figure 1Aⓑ) and reducing the absorbance intensity at 450 nm (Figure 1B). Interestingly, the results from the present study using citrate-capped AuNPs are opposite to the previous finding that I^−^ can inhibit the activity of casein-functionalized AuNPs [50]. A reasonable explanation for this different behavior is that the capped substances on the surface of AuNP affect the nanozyme core, leading to differences in the enzyme activity of nanozyme. We also found that biothiols such as cysteine was able to inhibit the I^−^-mediated oxidation of TMB when combined with either AuNP or I^−^ alone (Figure 1A(ⓓ,ⓕ)). These results implied that even when I^−^ dissociated from the surface of the nanoparticles, their peroxidase-mimicking activities were hampered by cysteine molecules that had not yet bound to the surface of AuNPs in solution. Overall, this system is expected to perform favorably in the detection of cysteine.

**Figure 2:**
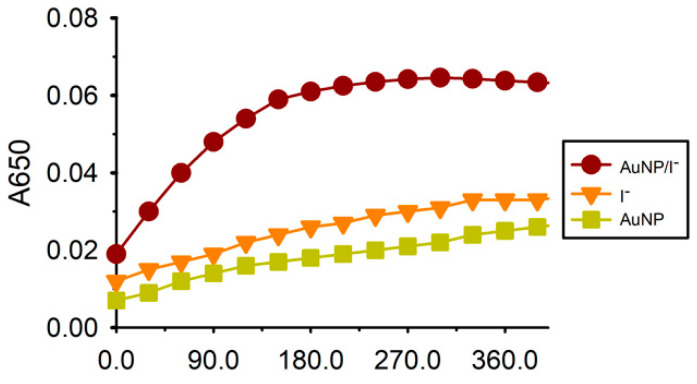
The time-absorbance curve of the TMB chromogenic reaction catalyzed by AuNP/I^−^, I^−^, and AuNP in 6 min.

In addition, to verify that the synergy of I^−^/AuNP improved the peroxidase-like activity, I^−^, AuNP, and I^−^/AuNP were tested before the addition of a stop solution. As the result shows in Figure 2, when the AuNP and I^−^ coexisted in the reaction solution, the absorbance at 650 nm increased rapidly with time. The absorbance was linear in the initial 120 s, so 120 s was selected as the initial rate period. Thus, the I^−^-modified AuNP had the fastest rate of 2.3 × 10^−4^/s for TMB oxidation, 3.5- and 3.9-fold higher than that of the I^-^ alone (6.7 × 10^−5^/s) and citrate-capped AuNP nanozyme (6.0 × 10^−5^/s), respectively, indicating I^−^ enhanced the activity of the AuNP.

### 3.2. Optimization of Experimental Conditions 

To improve the performance of the assay, the sensing conditions were optimized, including the concentrations of I^−^ and the volume of AuNPs. As shown in Figure S2A, the maximum ΔA was reached at an I^−^ concentration of 20 μM. Figure S2B shows that the highest catalytic activity was obtained when 10 μL of AuNP was added to the reaction solution. Next, the peroxidase-like activity of AuNPs was evaluated at different concentrations of H_2_O_2_, TMB, and HCl. After testing a range of H_2_O_2_ levels in the reaction medium (50 to 400 mM), the AuNPs showed the best catalytic activity at a concentration of 200 mM H_2_O_2_, as shown in Figure S2C. In addition, the optimal concentrations of TMB (Figure S2D) and HCl (Figure S2E) were 150 μM and 14.4 mM, respectively. Good activity that remained mostly stable was achieved using a reaction time of 2.5 min (Figure S2F). Accordingly, 2.5 min was chosen as the optimal reaction time for this method.

### 3.3. Colorimetric Sensing of Cysteine

**Figure 3:**
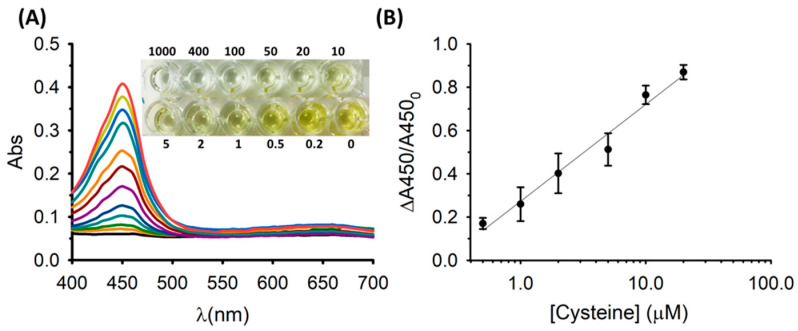
(**A**) Absorption spectra of the proposed assay with different concentrations of cysteine. The inset shows the color responses of iodide-capped AuNPs to different concentrations of cysteine. (**B**) Linear relationship between the normalized absorbance value at 450 nm and the concentration of cysteine in the buffer system.

The sensitivity of our system was determined in the presence of different concentrations of cysteine. To this end, assays were performed on the inhibition of I^−^ attachment to the surface of AuNPs by cysteine molecules in solution. It is worth noting that in the absence of cysteine, the I^−^ ions in the solution are directly absorbed onto the interface of the AuNPs, resulting in a significant change in the catalytic ability of the nanoparticles. The absorbance intensity of this assay at 450 nm was found to decrease, and the color of the solution gradually changed from yellow to colorless with an increasing cysteine concentration (Figure 3A). Under optimal conditions, a linear relationship was observed from 0.5 to 20 μM in a log scale. According to the 3σ/slope rule (σ shows the standard deviations of the blank sample, which is 0.0001 μM, while the slope is 0.448), the detection limit was determined to be 0.5 μM (Figure 3B). This low limit of detection was better than that obtained using fluorescence [51,52], electrochemical [53,54], or surface plasmonic detection [55,56] methods. The sensitivity of this strategy was lower than that of high-performance liquid chromatography (HPLC) [57,58], but the assay using this nanozyme was faster than HPLC analysis.

**Figure 4:**
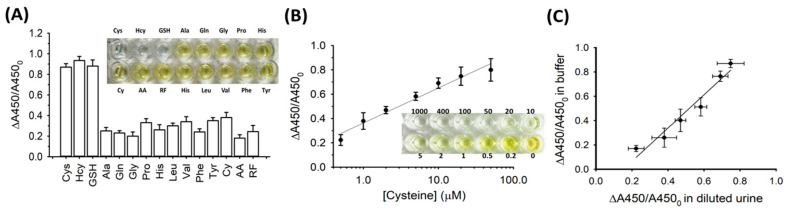
(**A**) Responses of our system toward 50 μM biothiols molecules including cysteine (Cys), homocysteine (Hcy), and glutathione (GSH) and other amino acids. The inset shows the corresponding color. (**B**) A plot of the normalized absorbance values at 450 nm of the proposed system versus the concentration of cysteine in the urine sample. The inset shows the color responses of iodide-capped AuNPs to different concentrations of cysteine. (**C**) Correlation between the normalized absorbance results obtained in the diluted urine samples and the standard samples of cysteine with six concentrations (0.5, 1, 2, 5, 10, 20 μM) (*R*^2^ = 0.95).

Selectivity is an important issue when designing biosensors for the recognition of targets. Thus, we tested the most abundant amino acids in the human body, including alanine (Ala), glutamine (Gln), glycine (Gly), proline (Pro), histidine (His), leucine (Leu), valine (Val), phenylalanine (Phe), and tyrosine (Tyr). In addition, cystine (Cy) which is the disulfide form of cysteine and other small molecules such as ascorbic acid (AA) and riboflavin (RF) were also tested. As indicated in Figure 4, only thiol-containing molecules including cysteine (Cys), homocysteine (Hcy), and glutathione (GSH) caused a noticeable decrease in absorption compared to the other thiol-free species. The inset of Figure 4 shows the corresponding color contrast and indicates that none of the molecules except thiol-containing species influenced the catalytic state of the AuNPs. Although the assay showed good selectivity for the analysis of biothiols in this study, AA is a common interferent that often interferes with urinalysis results. Ascorbic acid was found in 22.8% of urine specimens which had an average concentration of ~2000 uM [59]. Thus, to eliminate the interference of ascorbic acid, avoiding additional consumption of ascorbic acid at least 10 h before urinalysis is the recommended method [60].

To verify the feasibility of our system in practical applications, the proposed assay was used in the testing of biological fluids. The direct determination of cysteine in biological fluids, such as human urine, is significant for the early screening of cystinuria. To this end, we tested human urine samples for different concentrations of cysteine using the system established in this paper. Acetonitrile was added to the samples of normal human urine. Next, the urine samples were centrifuged to remove the acetonitrile-induced protein precipitation. The cysteine-spiked urine samples were prepared by adding cysteine to the urine samples at various concentrations. The detection limit was determined to be 0.5 μM based on the 3σ/slope rule (σ shows the standard deviations of the blank sample, which is 0.0001 μM, while the slope is 0.289) as shown in Figure 4B. A logistic relationship was observed between the cysteine levels, ranging from 0.5 to 50 μM. Figure 4C exhibited the correlation results between the detections of cysteine in urine samples and in standard samples. The linear relation had a slope of 1.37 with a correlation coefficient (R^2^) was 0.95, indicating that this system is a good candidate for use in the testing of real samples for cysteine detection.

## 4. Conclusions

In summary, we have established a tunable I^−^-capped AuNP surface that shows a changeable peroxidase-mimicking activity in response to biothiols. The catalytic behavior of AuNPs is inhibited by surface-absorbed cysteine molecules, which offers a versatile basis for biodetection. As a result, we developed a colorimetric sensor for the sensitive detection of biothiols using iodine-enhanced catalytic properties of AuNPs. This assay showed good specificity for the detection of biothiols against other non-specific compounds and was successfully applied for the testing of diluted human urine samples. Taken together, our results indicate the significant potential of this assay as a simple method for the detection of biothiols in biological fluids.

## Supplementary Materials

The following are available online at https://www.mdpi.com/2079-6374/10/9/113/s1. Figure S1: Difference in the fluorescence intensity of FITC-AuNPs before and after the addition of I^−^. Figure S2: Responses of AuNPs to different conditions.
