# Supplementary material for: Enhancement of the Peroxidase-Like Activity of Iodine-Capped Gold Nanoparticles for the Colorimetric Detection of Biothiols
Source: Biosensors (Basel). 2020 Sep 1;10(9):113. doi: 10.3390/bios10090113 (PMC7558680; doi:10.3390/bios10090113)
Supplement: Supplementary file 1 [file biosensors-10-00113-s001.pdf]

Supplemental information

Enhancement of the peroxidase-like activity of iodine-capped gold nanoparticles for the colorimetric detection of biothiols

Chia-Chen Chang [1,*], Tsz-Lian Hsu [2], Chie-Pein Chen [2,3] and Chen-Yu Chen [2,4*]

[1] Department of Medical Biotechnology and Laboratory Science, Chang Gung University, Taoyuan City, Taiwan
[2] Department of Obstetrics and Gynecology, Mackay Memorial Hospital, Taipei, Taiwan
[3] Department of Medical Research, MacKay Memorial Hospital, Taipei, Taiwan
[4] Department of Medicine, Mackay Medical College, New Taipei City, Taiwan
* Correspondence: chang@mail.cgu.edu.tw (C.C.Chang), f122481@mmh.org.tw (C.Y.Chen);

Analysis of iodide using FTIC-AuNPs

A solution of FITC-AuNPs was prepared according to a previous study. Briefly, 1 µl of 0.5 mM FITC was added to 1000 µL citrate-capped AuNPs. This mixture was equilibrated at 4°C for 24 h. After 24 h, 20 µL of FITC-capped AuNPs were mixed with 20 µL of different concentrations of I^-. The final volume of the resulting mixture was increased to 100 µL through the addition of ultrapure water for 30 min. The fluorescence intensity was monitored at 525 nm (excitation at 495 nm) using a SpectraMax M2 microplate fluorescence reader (Molecular Devices, Sunnyvale, CA, USA).

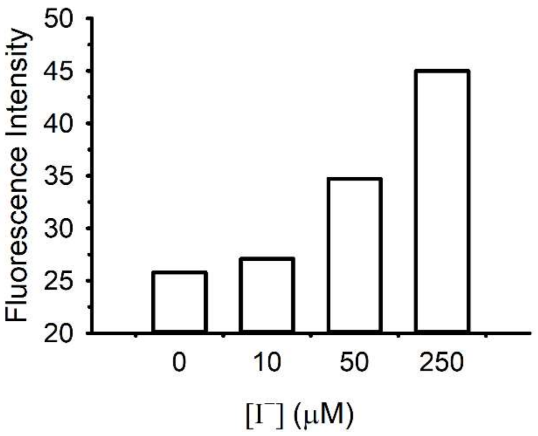

Figure S1. Difference in the fluorescence intensity of FITC-AuNPs before and after the addition of I^-.

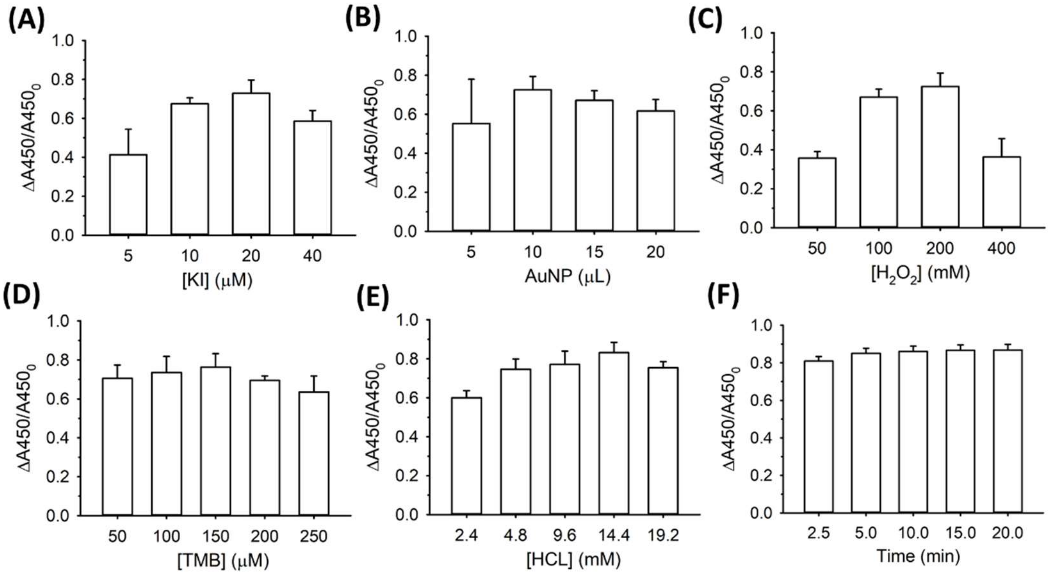

Figure S2. (A) Effect of the volume of AuNPs on the detection of 50 μM cysteine. Effect of the concentration of (B) TMB, (C) I^-, (D) HCl, and (E) H_2O_2 on the normalized absorbance ratio($\Delta A450/\ A450_0$). (E) Effect of reaction time; $\Delta A450 = A450_0 - A450_x$. $A450_0$ and $A450_x$ correspond to the absorption intensity of the reaction solution in the absence and presence of cysteine, respectively.
